# Minding the gap: learning and visual scanning behaviour in nocturnal bull ants

**DOI:** 10.1242/jeb.242245

**Published:** 2021-07-19

**Authors:** Muzahid Islam, Sudhakar Deeti, J. Frances Kamhi, Ken Cheng

**Affiliations:** 1 Department of Biological Sciences, Macquarie University, Sydney, NSW 2109, Australia; 2 Neuroscience Department, Oberlin College, Oberlin, OH 44074, USA

**Keywords:** Gap learning, Visual navigation, Success rate, Scene familiarity

## Abstract

Insects possess small brains but exhibit sophisticated behaviour, specifically their ability to learn to navigate within complex environments. To understand how they learn to navigate in a cluttered environment, we focused on learning and visual scanning behaviour in the Australian nocturnal bull ant, *Myrmecia midas*, which are exceptional visual navigators. We tested how individual ants learn to detour via a gap and how they cope with substantial spatial changes over trips. Homing *M. midas* ants encountered a barrier on their foraging route and had to find a 50 cm gap between symmetrical large black screens, at 1 m distance towards the nest direction from the centre of the releasing platform in both familiar (on-route) and semi-familiar (off-route) environments. Foragers were tested for up to 3 learning trips with the changed conditions in both environments. The results showed that on the familiar route, individual foragers learned the gap quickly compared with when they were tested in the semi-familiar environment. When the route was less familiar, and the panorama was changed, foragers were less successful at finding the gap and performed more scans on their way home. Scene familiarity thus played a significant role in visual scanning behaviour. In both on-route and off-route environments, panoramic changes significantly affected learning, initial orientation and scanning behaviour. Nevertheless, over a few trips, success at gap finding increased, visual scans were reduced, the paths became straighter, and individuals took less time to reach the goal.

## Abstract (precis)

**Summary:** Investigation of how nocturnal bull ants learn to move around obstacles in familiar and semi-familiar environments reveals that scene familiarity plays a significant role in navigation.

## INTRODUCTION

In a complex environment, animals exhibit an enhanced capacity to learn from individual experience (Bond et al., 2007; Chittka and Niven, 2009; Barron et al., 2015; Arena et al., 2017; Freas et al., 2019; Webb, 2019). Learning can open up new opportunities in spatial navigation, individual recognition, acquisition of new motor skills and association of multimodal cues with resources. Some insects, particularly bees and ants, readily acquire memories of the surrounding panorama and perform actions associated with those panoramas to navigate between their nests and a foraging site (Collett and Collett, 2002; Lent et al., 2013). Learning a novel visual task depends on behavioural flexibility in processing current visual stimuli combined with memories acquired through experiences (Schiffner et al., 2014; Giurfa, 2015; Ong et al., 2017). Solitary foraging ants rely on a number of sensory modalities and integrate this information to navigate (Hoinville and Wehner, 2018; Buehlmann et al., 2020).

The ability to move around or through gaps in obstacles is crucial for animals that navigate in cluttered environments. In the natural habitat, trees, bushes and shrubs create obstacles, presenting a series of apertures through which animals need to move (Kohler and Wehner, 2005; Mangan and Webb, 2012; Kabadayi et al., 2018). Budgerigars, *Melopsittacus undulatus*, trained to fly through a corridor between barriers, were able to do so efficiently when the gap was wide; when traversing narrower gaps, flight was interrupted but the birds passed through by changing their movement, indicating excellent perception of gaps (Schiffner et al., 2014). In another experiment in birds, wild rock doves, *Columba livia*, were able to navigate through a gap between horizontal obstacles with remarkable success using motion perception and optic flow (Ros et al., 2017). A study on gap learning in domestic dogs, *Canis familiaris*, showed that most could solve the detour task and find a gap in their first trial; over trials, they improved their detour ability (Osthaus et al., 2010). Other experiments showed that the majority of domestic dogs tended to replicate their previous successful learning trips, but it was more challenging for the more experienced dog to adapt to a novel path again in their subsequent trips (Pongrácz et al., 2001, 2003). A few recent studies have analysed insects such as bumblebees and honeybees in minimally cluttered environments (Baird and Dacke, 2016; Ong et al., 2017; Ravi et al., 2019; Burnett et al., 2020). These studies indicated that when individuals were confronted with obstacles with various spacing, insects chose the larger gap (Ong et al., 2017; Ravi et al., 2019) and used a brightness-based strategy for choosing among the different gaps (Baird and Dacke, 2016). A few studies have observed obstacle navigation to investigate learning and memory in ants in minimally cluttered environments (Luo et al., 2019; Wystrach et al., 2020). 

When ants are in unfamiliar environments, they look back toward the nest occasionally. This behaviour has been shown in Australian desert ants (*Melophorus bagoti*) during back-and-forth runs on their way to a previously unvisited foraging site (Freas and Cheng, 2018). Such runs mean that the ants look back towards the nest at multiple points on the route away from the nest. It has been suggested that these turn-back-and-look behaviours are necessary for learning panoramic views and then generalising to other sites (Zeil, 2012; Zeil et al., 2014; Fleischmann et al., 2016, 2017; Freas and Cheng, 2018; Fleischmann et al., 2018; Deeti et al., 2020). Recent studies showed that scanning behaviours play a crucial role in the navigation of ants. Before starting to forage, *Cataglyphis* and *Myrmecia* ants perform learning walks in which they stop and turn back towards the nest occasionally (Fleischmann et al., 2017, 2018; Jayatilaka et al., 2018). In experienced foragers, scanning behaviour is performed at a higher rate in situations where the surrounding panorama was unfamiliar to the ants, suggesting that scanning contributes to visual learning (Wystrach et al., 2014; Philippides et al., 2011; Zeil et al., 2014). Like ants, other animals, including bees, wasps, lizards, birds, fishes, rats and monkeys, perform head movements when they encounter any surrounding changes or environmental unfamiliarity (Ros et al., 2017).

Several studies on visual navigation in the genus *Myrmecia* have shown that they use both the terrestrial panorama and sky compass cues to navigate between the nest and foraging sites (Reid et al., 2011; Zeil et al., 2014; Freas et al., 2017; Freas and Cheng, 2019). When the surrounding panorama was changed by a small (*Myrmecia pyriformis*) or large amount (*M. midas*), the navigational efficiency of the ants was disrupted (Islam et al., 2020; Narendra and Ramirez-Esquivel, 2017; Freas et al., 2018). In *M. pyriformi*s, a nocturnal species, foragers showed major disruption in their navigational efficiency after a subtle change of the surrounding panorama caused by the removal of three trees on their foraging corridor (Narendra and Ramirez-Esquivel, 2017). On the first trip, foragers were less directed, showed reduction of their speed and foraging success, and increased the number of scans. Moreover, these behavioural changes persisted over multiple nights before the ants returned to their natural foraging behaviours (Narendra and Ramirez-Esquivel, 2017). The effects of a large panorama change on another nocturnal bull ant *M. midas* were studied when construction work resulted in the addition of a fence and the removal of several trees near the nest site. When foragers were displaced locally off-route after these changes, they were initially unable to orient correctly to the nest direction and their return paths were less straight, suggesting increased navigational uncertainty (Islam et al., 2020). Continued testing showed that foragers recovered in 2–7 days (one trip per day) in both initial orientation and path straightness. Note that the *M. midas* ants tested in Islam et al.’s (2020) study consisted of those that arrived at their foraging tree, and thus were already successful at outbound navigation, while the *M. pyriformis* examined in Narendra and Ramirez-Esquivel's (2017) study consisted of those emerging from their nest. No research on gap learning in the natural habitat has yet examined performance trip by trip over repeated trials in any ant. The current research investigated in *M. midas* (a) gap perception and learning in familiar (on-route) and semi-familiar (off-route) contexts and (b) visual scanning behaviour with changes of the visual panorama, including the influence of scene familiarity on scanning.

## MATERIALS AND METHODS

### Study animals

Cryophilic, nocturnally foraging bull ants, *Myrmecia midas* Clark 1951, are found mainly along the coastlines of several eastern Australian states. The vegetation around the nest areas contains many eucalyptus trees. The landscape around the nests at our test site was a wooded area with grass and leaf litter on the ground. The ants usually nest close to the base of a eucalyptus tree (<30 cm) and, in summer, some portion of the ants forages on the tree located at the nest during the evening twilight, called the nest tree. Worker ants also forage on trees around the nest, mostly a nearby tree, called a foraging tree (Freas et al., 2017). Each forager primarily travels to the same foraging tree each night. The foraging activity of bull ants starts just after sunset, in the evening twilight, and ants come back to their nest before morning. Australia does not have ethical requirements concerning work with ants. The experimental procedures were non-invasive and, as in previous behavioural research with these ants (Freas et al., 2018; Freas and Cheng, 2019), they had no notable adverse effects on the individuals or their colony.

### Experimental setup

Two experiments were conducted at nests on the northern portion of Macquarie University's North Ryde campus in Sydney, Australia (33°46′11′′S, 151°06′40′′E), from November 2018 to March 2019. From the chosen nests, the ants that were foraging on a particular tree were captured in the evening twilight (20:00–21:00 h) at the base of their foraging tree using foam-stoppered transparent glass tubes. In nest A, foragers were collected from a foraging tree, which was located 9 m away from the nest, and in nest B, foragers were collected from a foraging tree 8 m away. The collected ants were cooled down for 5 min in an icebox and painted on their body with a specific colour code (Tamiya brand) for individual identification; ants were then transferred back into the test tube. They recovered within 10 min and were fed with honey water with the help of a Pasteur pipette and stored in a dark room overnight. The next morning, between 07:00 h and 09:30 h, ants were tested in different experimental conditions.

At the nest site, a week before the experimentation began, we cleared the vegetation on the ants’ foraging corridor in nest A and also cleared the vegetation both along the foraging corridor and up to 10 m in the direction 90 deg clockwise off route in nest B. In nest A (area 9×4 m), 1 m squares were gridded between the nest and foraging tree using synthetic white string and tent pegs. In nest B, we made a 9×4 m grid with 1 m squares in the off-route direction.

**Fig. 1.**
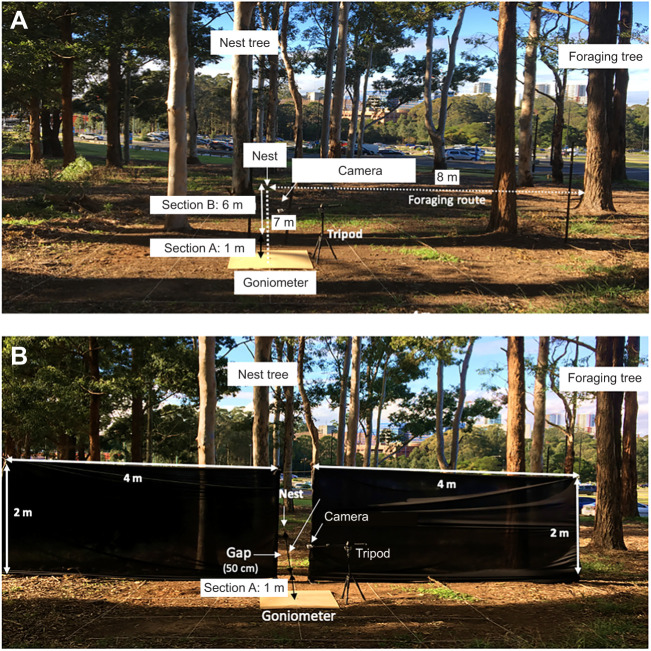
**Experimental set up for examining gap learning in *Myrmecia midas* ants in a semi-familiar environment.** (A) The control condition where a goniometer was placed 7 m away from the nest, and 90 deg clockwise from the natural foraging route (experiment 2). (B) The gap position and symmetrical screens on either side on the return journey of displaced foragers. In both cases, a video camera was fixed just above the displacement centre to record initial heading of foragers on the goniometer.

To obtain precise tracks of the foragers during the experiment, and to note initial heading directions, we placed a wooden goniometer at ground level at the release site (Fig. 1). The wooden platform was 50 cm in radius with 24 equal, 15 deg wedges drawn on it and a circular indentation in the centre with a 15 mm diameter and 25 mm depth for releasing the ants into. We released the foragers singly on the centre of the goniometer and covered the hole with a plastic lid with the help of black tape to settle the ants for at least 30 s before starting the test. The initial headings of the foragers’ first crossings at 50 cm (the wedge that was crossed) were considered as their heading direction.

### Experiment 1: gap perception in familiar environment

Two separate groups of individually painted ants from nest A were used in this experiment: one for the control condition (*n*=24) and one for the gap-learning condition (*n*=32).

#### Control condition

In the control condition, ants were released on the goniometer, placed between the nest and the foraging tree, 7 m away from the nest, and were allowed to return home. The initial headings of the foragers’ first crossings at 50 cm from the centre of the goniometer were recorded by the video camera (GoPro Black Hero 7 Action Camera, 2.7K, 60 frames s^−1^) and also noted on a piece of graph paper. We also tracked the ants’ entire paths on the gridded paper from the release point to their nest and noted the number and position of scans on their way home. We divided the foraging route into two sections: section A, the first metre from the release point to the middle of the gap between the screens; and section B, the rest of the 6 m path from the middle of the gap to the nest. We allowed a maximum of 4 min in section A and 10 min in section B for individual ants to reach their nest. Each of the individually marked ants was tested for three consecutive trials, once per day. At the end of each trial, each ant was allowed to enter its nest. They were captured on their next appearance at the base of the foraging tree on subsequent nights.

#### Gap-learning condition

To make a gap and block the familiar view towards the nest, we constructed two symmetrical large black screens (4 m×2 m) at 6 m from the nest on the ants’ foraging route, which were thus located 1 m from the goniometer centre in the nest direction. Between the black screens there was a 50 cm gap. The gap intersected a line connecting the nest, the centre of the wooden goniometer and the middle of the foraging tree. Ants were again released on the centre of the goniometer placed 7 m from the nest but only 1 m from the symmetrical black screens and were allowed to return home. We recorded as initial headings the foragers’ first crossings at 50 cm (the wedge that was crossed). We also recorded whether the ant succeeded in navigating through the gap throughout the duration of the trip. To do so, we allowed the foragers up to 4 min from emergence on the platform to reach the gap from the centre of the goniometer. Foragers were considered successful if they found the gap 1 m ahead of the release point within 4 min of starting their journey from the goniometer centre (section A). We recorded the number and position of the scans before and after finding the gap and again tracked the ants’ paths until they reached their home. One observer tracked the ants’ paths from the centre of goniometer to the gap 1 m away. A second observer tracked the paths after an ant crossed the gap until she reached the nest. If an ant failed to find the gap within 4 min, the trial was considered unsuccessful, and the ant was captured and released between the black screens (at the gap) to travel to her nest. It was ensured that all tested ants got back to their nest after each test. We allowed a maximum of 10 min for travel from the gap to their nest (section B). Each ant was collected at the base of the foraging tree on a subsequent night to be tested 3 times in this condition, once per day. The screens were taken down after the experimentation of each day.

### Experiment 2: gap perception in semi-familiar environment

Two separate groups of individually painted ants from nest B were used in this experiment: one for the control condition (*n*=26) and one for the gap-learning condition (*n*=34).

#### Control condition

Similar to the control condition of experiment 1, foragers were collected at the foraging tree base and released the next morning. However, in this experiment they were released at 7 m from the nest in a direction 90 deg clockwise from the foraging tree (Fig. 1). Ants were again placed on the goniometer and allowed to run home. We quantified the compass direction of their headings by filming them with the video camera. The initial headings of the foragers’ first crossings at 50 cm were noted. We again tracked the ants’ paths on gridded paper along with the positions of scans. Each ant was again tested for three consecutive trials, once per day. At the end of each test, the ant was allowed to enter its nest. Ants were captured on their next appearance at the bottom of the foraging tree in the evening twilight on subsequent nights.

#### Gap-learning condition

In the gap-learning condition, on the off-route, two symmetrical black screens (4 m×2 m) were placed 6 m from the nest position and 1 m from the goniometer centre in the nest direction. Again, there was a 50 cm gap between the black screens, aligning the nest, the gap and the centre of the goniometer. Each ant was again released on the centre of the goniometer and allowed to run home. We recorded as initial headings the foragers’ first crossings at 50 cm (the wedge that was crossed). We recorded whether the forager was successful by the same criterion as in experiment 1 along with the duration of the entire trial. Again, we recorded the number and positions of scans before and after finding the gap, thus following the same procedure as in experiment 1. The screens were taken down at the end of each day's experimentation. Each ant was tested 3 times, once per day.

### Filming and 360 deg photo-capturing procedure

To obtain information about the initial behaviour and heading of ants, we placed a Go Pro Black Hero-7 camera directly above the goniometer. We fixed the camera using a horizontal arm which extended from the top of a tripod. The camera was focused straight down during all of the conditions, filming a 100 cm×100 cm area of the wooden platform (goniometer). Ants were placed in the indentation at the centre of the goniometer to start a test. We started filming when an ant was coming out from the centre of the goniometer and stopped when the ant crossed the 50 cm radius line from the centre or 5 min after an ant emerged, if the ant never reached the 50 cm radius line. We recorded the initial orientation of individual foragers on all three learning trips or control trips at 2.7K and 60 frames s^−1^. A Bloggie camera (MHS-PM5, Sony Co.) was used to obtain a panoramic view of release points in different conditions. The 360 deg panoramic photos were taken at the centre of the goniometer where the foragers were released in all four conditions across the two experiments.

### Statistical procedure

From the video records and the recorded paths on paper, we calculated the following measures as dependent variables.

#### Path straightness

Path straightness was calculated as the ratio between the total path length of individual foragers and the straight-line distance from the release point to the 50 cm circumference (i.e. 50 cm). The range of path straightness was transformed to span 0 to 0.5.

#### Duration on goniometer

We calculated the duration from the time a forager came out from the centre of the goniometer to the time that it crossed the 50 cm radius. When foragers stopped for more than 10 s in the same place, we considered this duration as a resting period, and reduced it to 1 s.

#### Scans

We observed foragers stopping and scanning the environment by turning on the spot, before walking towards their chosen direction as well as en route. A scan is a saccadic movement with a pause after it. If the extent of heading rotation of a forager was between 1 and 360 deg, that was considered as a single scan.

#### Scanning zones

In the control condition, more than 90% of scans were performed within 15 cm of the centre of the goniometer, before the ants chose a heading direction. We considered this zone as the start zone. The rest of space was considered as the route zone.

The goniometer data were analysed with circular statistics (Batschelet, 1981) using the circular statistics software Oriana Version 4 (KOVACH Computing Service). To examine the foragers’ initial orientation, Rayleigh's tests were conducted, testing whether data met the conditions of a uniform distribution (*P*>0.05) or whether the distribution of headings was non-randomly distributed. If the data were non-uniform, V-tests were conducted to determine whether the distribution of initial heading directions was significantly clustered in the nest direction. We also examined whether the nest direction fell within the 95% confidence intervals of the mean vectors of heading distributions. We used the digitizing software Graph-Click (https://graphclick.en.softonic.com/mac), for digitizing the paths of individual foragers. A custom-written MATLAB (MATLAB 2019b) program was used to plot the paths of the foragers and measure the path straightness of individual foragers in all conditions. For the control conditions, the straight-line distance for individual foragers was 7 m. In the gap-learning conditions, we divided the path into two sections: section A, from the release point to the gap, and section B, from the gap to the nest. For path straightness in both experiments, we conducted repeated-measures ANOVA to compare across the third control run in the control condition and the three trips of the gap-learning condition. In experiment 2, ANOVA were conducted separately for path straightness in section A and section B. Each learning trip in the gap-learning condition was compared with control run 3, using a Bonferroni correction. We did not observe any notable differences between the control runs, so we chose only the third control trip to compare with the other conditions.

In section A, foragers’ duration was timed when individual ants started their journey from the release point until they reached the gap between the black screens (or where the screens would be) in both the control condition and the gap-learning condition. In section B, foragers’ duration was calculated from the point when they either crossed the gap (or where the gap would be) or were released at the middle of the gap until they reached the nest location. Statistical tests using ANOVA were conducted using the same statistical procedure as that used for path straightness.

## RESULTS

### Gap-finding success rate

**Fig. 2.**
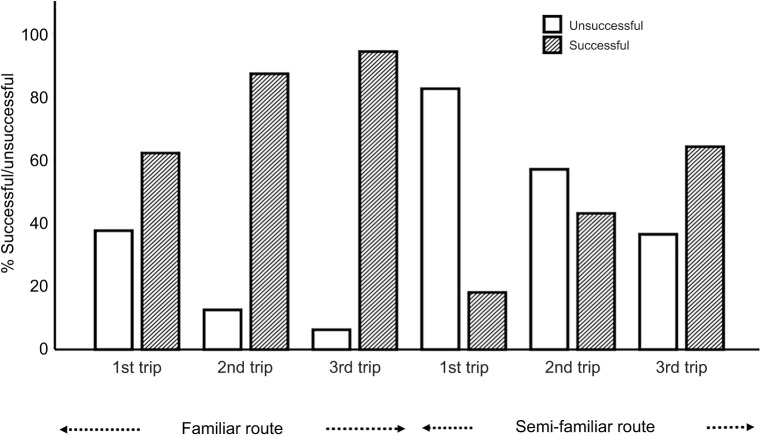
**Success rate of individual foragers during gap learning with changes of surrounding panorama.** Data are for the familiar (experiment 1) and semi-familiar (experiment 2) environments.

All of the foragers were successful at finding the gap when we released them on the goniometer, which was 7 m away from the nest, on both the familiar and semi-familiar routes. When we made a gap and changed the front-side panorama using symmetrical black screens at 1 m distance from the release point, many foragers were unsuccessful at finding the gap in their first attempt (Fig. 2). In subsequent learning trips, foragers’ success rate increased. The success rate was higher in the familiar environment than in the semi-familiar environment in the gap-learning conditions.

### Initial heading direction

**Fig. 3.**
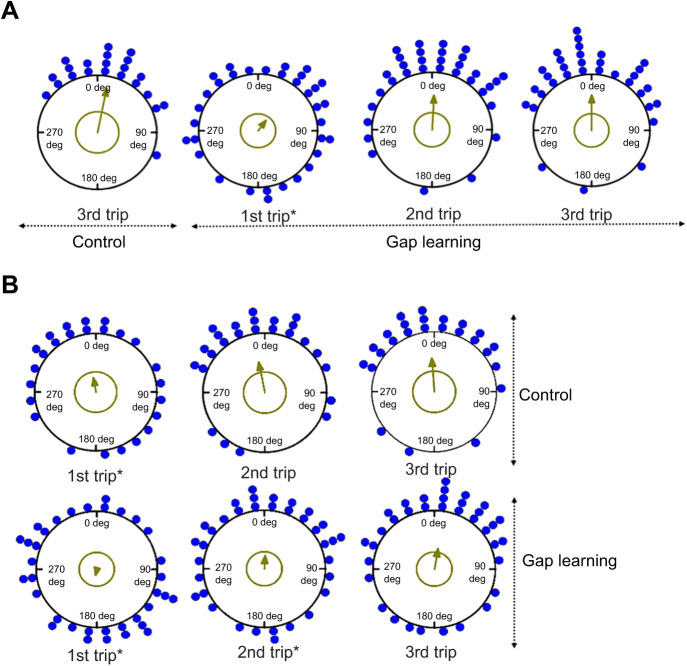
**Circular distributions of initial headings under the different conditions.** Forager directions are shown at 50 cm from the release point on (A) the familiar route (experiment 1) and (B) the semi-familiar route (experiment 2) in both control and gap-learning conditions. The nest direction for each figure is at 0 deg. The arrows denote the length of the mean vector of each condition. Asterisks indicate significant differences of initial heading compared with the (third) control trip.

When foragers emerged onto the goniometer platform, they usually stopped for a while and scanned before heading in a chosen direction. In the familiar-route control condition, foragers showed non-uniform movement at 50 cm from the release point (Table 1, Fig. 3A) and their orientations were in the nest direction at 0 deg (V-test, Table 1). When we changed the front-side panorama and made a gap in middle of the two screens (gap-learning condition), the first learning trip was uniform in the distribution of initial headings (Fig. 3A, Table 1). On trips 2 and 3, foragers again showed non-uniform initial headings and their orientations were significant in the nest direction at 0 deg (Table 1, Fig. 3A). In the semi-familiar environment, foragers struggled to find the correct heading direction. The first control trips showed a uniform distribution of initial headings (Table 1, Fig. 3B). However, in the second and third control trips, foragers’ initial orientation was non-uniform and significantly pointed in the nest direction (Table 1, Fig. 3B). In the semi-familiar gap-learning condition, initial headings were uniformly distributed on the first and second trips, and only on the third trip were the ants oriented significantly towards the nest (Fig. 3B, Table 1).

**Table 1.**
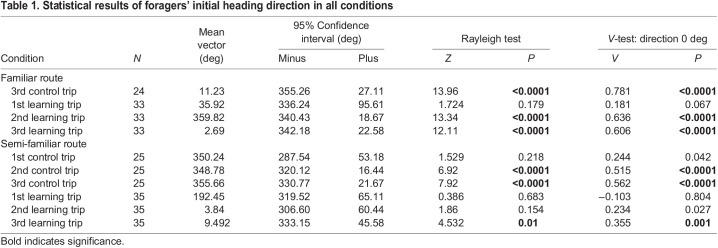
Statistical results of foragers' initial heading direction in all conditions

### Path straightness

**Fig. 4.**
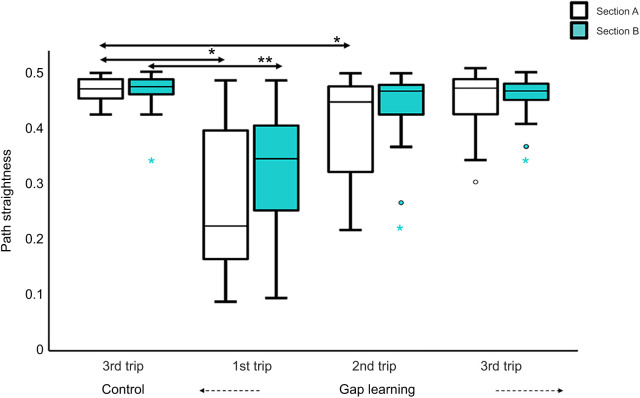
**Path straightness of foragers on the familiar route.** The box plots show path straightness for section A (1 m) and section B (6 m) separately in both control and gap-learning conditions for experiment 1. Asterisks (* and **) and arrows indicate significant differences of path straightness compared with the third trip of the control condition. Circles indicate exceptional path straightness outside of the box plot range. The box plots indicate medians (solid line), box margins (25th and 75th percentiles) and whiskers (5th and 95th percentiles) in this and all following figures. Asterisks represent significant differences (*section A and **section B).

**Fig. 5.**
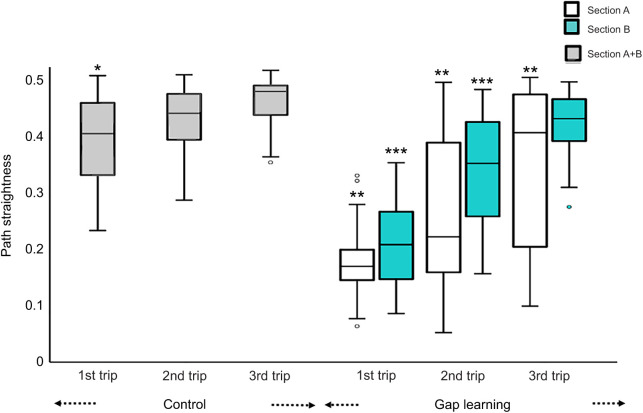
**Path straightness of foragers on the semi-familiar route.** The box plots show path straightness in both control and gap-learning conditions for experiment 2. For the control condition, path straightness was measured from the release point to the nest (section A+B=7 m); in the gap-learning condition, path straightness is shown separately for section A (1 m) and section B (6 m). Asterisks (*first trip of control condition, **section A and ***section B) indicate significant differences of path straightness compared with the third trip of the control condition.

In the control conditions of both the familiar and semi-familiar environments, foragers went straight to the nest. However, on the first gap-learning trips on both routes, foragers struggled to find the gap and meandered in both section A and section B. Over successive learning trips, foragers’ paths became straighter and meandered less (Figs 4 and 5). Path recovery was quicker on the familiar route than on the semi-familiar route. To test for differences in path straightness, repeated-measures ANOVA were conducted. On the familiar route, the results showed a significant effect of trips in both section A (*F*_3,93_=31.11, *P*<0.0001) and section B (*F*_3,93_=32.84, *P*<0.0001). Bonferroni-corrected *post hoc* tests revealed that the first and second learning trips of the experimental condition were significantly less straight compared with the third control trip in section A, while only the first trip was significantly less straight in section B. There was no significant effect on path straightness in section A+B of the semi-familiar control condition (*F*_2,48_=5.79, *P*=0.06). Path straightness differed significantly across trips on the semi-familiar route in both section A (*F*_2,66_=23.47, *P*<0.0001) and section B (*F*_2,66_=93.22, *P*<0.0001) in the gap-learning condition, with most of the trips being significantly less straight than in the third control trip (Fig. 5).

### Duration

**Fig. 6.**
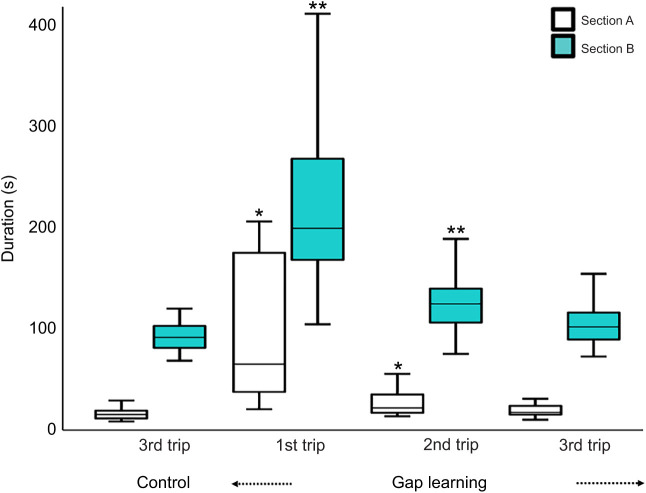
**Duration of foragers’ trips on the familiar route.** The box plots show duration for section A (1 m) and section B (6 m) separately in both control and gap-learning conditions for experiment 1. Asterisks (*section A and **section B) indicate significant differences of duration during homeward navigation compared with the third trip of the control condition.

**Fig. 7.**
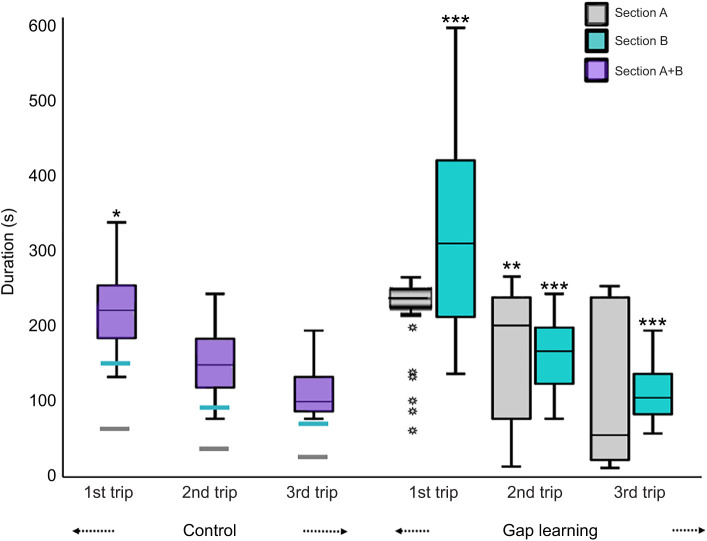
**Duration of foragers’ trips on the semi-familiar route.** The box plots show duration in both control and gap-learning conditions for experiment 2. For the control condition, the duration was measured from the release point to the nest (section A+B=7 m). In the gap-learning condition, duration is shown separately for section A (1 m) and section B (6 m). Asterisks (*first trip of control condition, **section A and ***section B) indicate significant differences of duration during homeward navigation compared with the third trip of the control condition.

In the control conditions of both the familiar and semi-familiar environments, foragers took less time to navigate from the release point to the nest compared with the learning conditions. However, on the first gap-learning trip on both kinds of routes, foragers took longer in both section A and section B. Over successive learning trips, foragers became faster at reaching the goal (Figs 6 and 7). Repeated-measures ANOVA were conducted to test for differences in duration. On the familiar route, the results showed a significant effect of trips in both section A (*F*_3,93_=26.45, *P*<0.0001) and section B (*F*_3,93_=75.12, *P*<0.0001). Bonferroni-corrected *post hoc* tests revealed that the duration of first trips and second trips was significantly longer than that of the third control trip in both sections (Fig. 6). On the semi-familiar route, in the control condition in section A+B, there was also a significant effect of trips on duration (*F*_2,48_=32.34, *P*<0.0001) (Fig. 7). Bonferroni-corrected *post hoc* tests revealed that the duration of the first control trip was significantly longer than that of the third control trip. In the gap-learning condition for the semi-familiar route, the duration was significantly different across trips in both section A (*F*_2,66_=34.28, *P*<0.0001) and section B (*F*_2,66_=64.41, *P*<0.0001), with most of the trips taking significantly longer compared with the third control trip (Fig. 7).

### Visual scanning behaviour

**Fig. 8.**
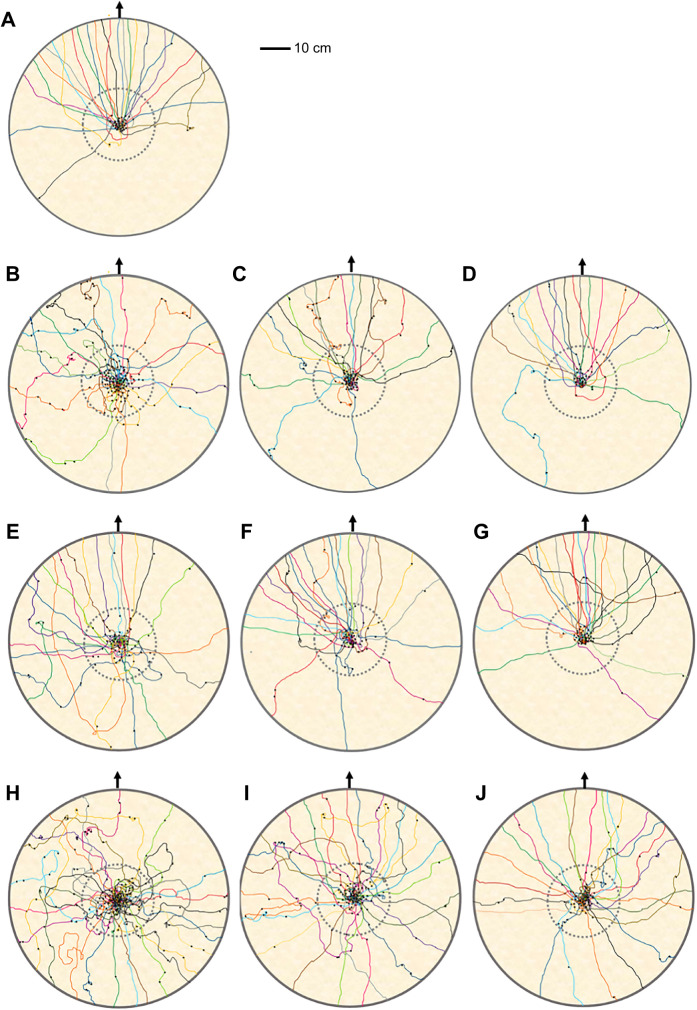
**Foragers’ path and scanning positions (black dots) on the goniometer in different conditions on the familiar and semi-familiar route.** (A–D) Proportion of scans in the ‘start zone’ (dotted circle, 15 cm radius) on the familiar route: (A) 3rd control trial 94%, (B) 1st gap-learning trip 63%, (C) 2nd gap-learning trip 78%, (D) 3rd gap-learning trip 84%. (E–J) Proportion of scans in the start zone on the semi-familiar route: (E) 1st control trip 74%, (F) 2nd control trip 82%, (G) 3rd control trip 91%, (H) 1st gap-learning trip 58%, (I) 2nd gap-learning trip 66%, (J) 3rd gap-learning trip 75%. Individual foragers were released on the centre of a wooden goniometer and the headings were recorded using a camera (see Materials and Methods). The solid circles indicate the goniometer border. The ‘route zone’ occupies all areas outside of the start zone on the goniometer. The black arrow indicates the nest direction. Each path colour represents an individual ant. In all of the trials of all conditions, foragers performed more scans in the start zone than in the route zone.

**Fig. 9.**
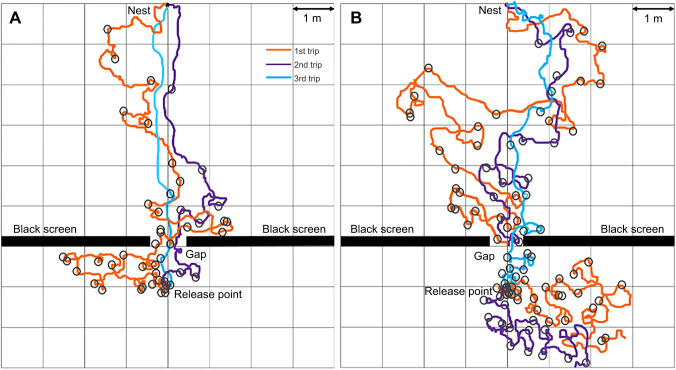
**Example paths of two foragers, one in each panel, from the release point to the nest in each experiment.** The circles show the positions of scans. If a forager did not find the gap within 4 min, we considered it ‘unsuccessful’ and released it in the middle of the gap and allowed it to return home. Over learning trips, the paths became straighter, scans became reduced, and foragers took less time to reach their nest. On the familiar route (A), the ant was successful in the three consecutive learning trips whereas on the unfamiliar route (B), the 1st and 2nd gap-learning trips were unsuccessful, but the 3rd gap-learning trip was successful.

In displacement experiments, foragers typically perform some immediate scans before heading off in a chosen direction (Fig. 8), but when foragers first encountered substantial changes in the surrounding visual panorama, on- or off-route, they performed a large number of scans, including scans along the route (Fig. 9). In control trips, foragers performed scans mostly just after coming out from the goniometer centre. In contrast, when foragers first encountered panoramic changes in both the familiar and semi-familiar environments, they performed many scans. Throughout the repeated trials, scanning was reduced in both section A and section B in both experiments (Figs 8 and 9; Fig. S1). The repeated-measures ANOVA revealed a significant effect of trips in section A in both the familiar (*F*_2,62_=33.58, *P*<0.0001) and semi-familiar environments (*F*_2,66_=32.84, *P*<0.0001). On both routes, the number of scans in the first and second gap learning trips was significantly different (*P*<0.05) from the third control trips (Fig. S1). Likewise, in section B, the same pattern occurred (*P*<0.05, Fig. 9; Fig. S1).

## DISCUSSION

In the present study, we asked how individual foragers learned to navigate through a gap and visual changes in their surroundings during homeward navigation. When displaced *M. midas* foragers encountered a barrier on their foraging route and had to find a 50 cm gap between symmetrical large black screens at 1 m distance towards the nest, the foragers on the familiar route were more efficient and successful in finding the gap compared with those off route (semi-familiar environment). When the route was less familiar and the panorama was changed, foragers had less success in finding the gap, performed more scans and the paths were less straight on their way home. Scene familiarity thus played a significant role in visual scanning behaviour. In the control condition, foragers performed scans mainly at the start of their journey, but with the visual panorama change, foragers performed a significant number of scans along their foraging route as well. Over their learning, foragers performed a smaller proportion of scans outside of the start zone. The number of scans was higher in section A (first 1 m) than in section B (the rest of the route). Overall, in both on-route and off-route environments, panoramic changes significantly affected initial orientation, learning and visual scanning behaviour of individual foragers. Nevertheless, on the third trip, success at gap finding increased, the paths of foragers became straighter, visual scans were reduced and individuals took less time to reach the nest.

In our experiments, the initial heading direction of foragers differed between the familiar and semi-familiar environments. In the familiar environment, foragers’ headings were nest directed after just a single experience with the panoramic changes, but in the semi-familiar environment, a substantial minority failed to find the gap even on the third learning trial. In vertebrate animals, several studies have shown gradual improvements of heading to find the gap in detouring over multiple trials (Smith and Litchfield, 2010; Boogert et al., 2011; Schiffner et al., 2014; Ros et al., 2017). For instances, gap perception studies in domestic dogs, *C. familiaris*, showed that most of the dogs could find a gap and solve the detour task in their first trial and improved their gap perception over trials (Osthaus et al., 2010). Among ants, the desert ant *M. bagoti* learned the panoramic visual cues on a single trip to a feeder (Freas and Cheng, 2018), with initial headings directed towards the nest. Another desert ant, *Cataglyphis velox*, however, took more than two experiences to orient correctly (Freas et al., 2019). In contrast, *M. pyriformis* foragers’ orientation was greatly affected by small changes along their foraging route (Narendra and Ramirez-Esquivel, 2017). Large (30%) panoramic changes disrupted the initial heading direction of homing *M. midas*, but foragers, at least those that managed to get to their foraging tree, recovered from the changes quickly (Islam et al., 2020). It is not known how outbound foragers (the population studied by Narendra and Ramirez-Esquivel, 2017) would have fared with the large panoramic changes. It is impressive that, in our current experiments, foragers learned the panorama and memorised the gap position in the familiar route so quickly even though it was challenging for them in off-route semi-familiar conditions, suggesting a critical role of scene familiarity for robust panorama learning and memory.

Scanning is a species-typical behaviour in *M. midas* foragers in which the ant suddenly stops and rotates on the spot, facing in different directions. *Melophorus midas* foragers rotated their head from 1 to over 360 deg during a single scanning bout*.* Typically, displaced foragers performed quick scans before heading off in a chosen direction, but when they encountered any substantial changes in the surrounding panorama, they performed a large number of scans, including scans along their navigational route. The scanning likely contributes to their learning and scene memory. Like nocturnal bull ants, Australian desert ants, *M. bagoti*, perform scans at the start of their trips, and when they experience a decrease in visual familiarity (Wystrach et al., 2014) or encounter aversive experiences (Wystrach et al., 2019, 2020). In our experiment, visual changes led to increasing scanning at the beginning, presumably in order to find the gap, but also along the route after passing the gap when foragers turned back to scan, presumably to learn the changes for future navigation. Previous studies with desert ants have shown that the foragers learn the panorama during back-and-forth runs on their way to reaching a foraging site (Wystrach et al., 2014; Fleischmann et al., 2018; Freas and Cheng, 2018). Such foragers look back towards the panoramic changes at multiple points on the route. It has been suggested that the turn-back-and-look behaviour is necessary for foragers to learn panoramic views and then to generalise to other sites (Nicholson et al., 1999; Zeil, 2012; Zeil et al., 2014; Fleischmann et al., 2016, 2017; Freas and Cheng, 2018; Fleischmann et al., 2018). Looking probably supports learning.

### Conclusion

*Myrmecia midas* foragers exhibit differences in gap learning as a function of scene familiarity. Ants were worse at learning off route than on route. When foragers encountered a new panorama with a gap in the middle for the first time, their initial orientation was not directed, and a small proportion of foragers were successful. Ants performed characteristic scans and meandering along the route. However, foragers become more successful over trips, taking less time, performing fewer scans and exhibiting straighter paths. These findings suggest that nocturnal bull ants learn to cope better and quicker with a scene change on their familiar route than in an off-route environment.

## Supplementary Material

Supplementary information
