# Supplementary material for: Minding the gap: learning and visual scanning behaviour in nocturnal bull ants
Source: J Exp Biol. 2021 Jul 19;224(14):jeb242245. doi: 10.1242/jeb.242245 (PMC8325935; doi:10.1242/jeb.242245)
Supplement: Supplementary information [file jexbio-224-242245-s1.pdf]

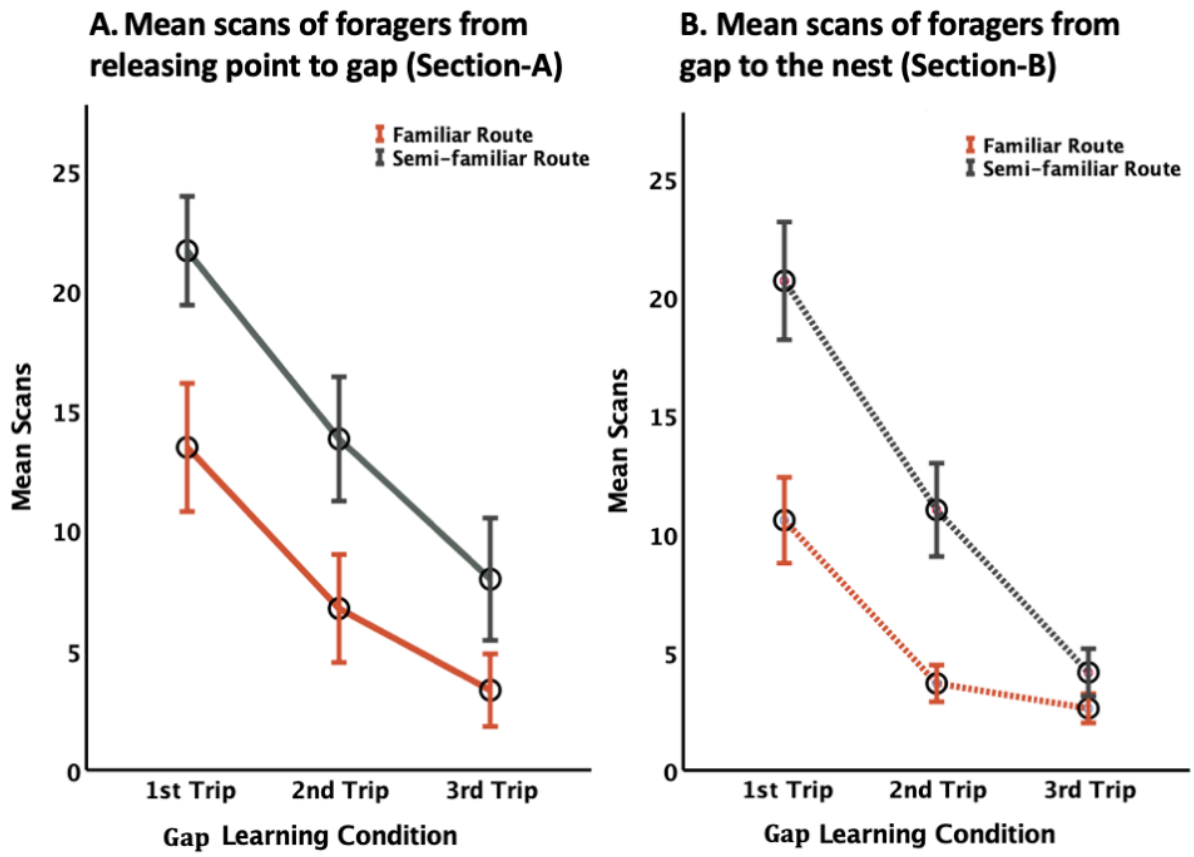

Fig. S1. Mean number of scan of foragers in Section-A and Section-B on Gap Learning trials in the Familiar and Semi-familiar environments. Error bars indicate the 95% confidential intervals about the mean.

Journal of Experimental Biology • Supplementary information
